# Predicting vaccine hesitancy among parents towards COVID-19 vaccination for their children in Singapore

**DOI:** 10.3389/fped.2022.994675

**Published:** 2022-10-10

**Authors:** Jia Ming Low, Chloe Wen Ting Soo, TA Phuong, Youjia Zhong, Le Ye Lee

**Affiliations:** ^1^ Department of Neonatology, Khoo Teck Puat - National University Children’s Medical Institute, National University Health System, Singapore, Singapore; ^2^ Department of Paediatrics, Yong Loo Lin School of Medicine, National University of Singapore, Singapore, Singapore

**Keywords:** COVID-19, paediatric, parental attitudes, vaccination, social media

## Abstract

**Background:**

There was a considerably slower uptake among children despite the high COVID-19 vaccination uptake amongst adults and adolescents in Singapore. This was concerning as unvaccinated children are at risk of severe COVID-19 infections and a source and reservoir of infections. We sought to understand the impact of social media on parental vaccine hesitancy and to determine the risk factors associated with vaccine hesitancy.

**Methods:**

An electronic survey conducted from November 2021 to March 2022. Data on the demographic profiles of respondents and to classify them based on their vaccine hesitancy status. Data including the choice of social media used to obtain information on the COVID-19 pandemic, frequency of use were collected. Statistical significance was defined as *p* < 0.05.

**Results:**

Six hundred and twenty-eight parents participated. 66.9% of parents were not vaccine hesitant. About a third (27.2%) considered themselves somewhat vaccine hesitant. Fathers were more vaccine hesitant than mothers. Vaccine hesitancy was also associated with having a lower household income, unvaccinated parents, knowing someone with an adverse reaction to the Covid 19 vaccine and having a low level of trust in their child's doctor. There was no significant difference with high usage of social media between parents who were not vaccine hesitant vs. those who were vaccine hesitant. Despite high usage of social media, about two thirds (62.7%) of parents preferred print material to obtain COVID-19 related information. Parental trust in their child's doctor was the most significant factor in determining vaccine hesitancy amongst parents. When the variables of gender, household income status, vaccine status were further analysed with a multinomial logistic regression model, vaccine hesitancy in a parent could be predicted with a 70% accuracy, and non-vaccine hesitancy with a 92.4% accuracy.

**Conclusion:**

Newspapers and print media were the primary sources used in obtaining information on COVID-19 vaccine safety and efficacy, especially amongst parents with a higher household income. Healthcare providers should continue to establish rapport amongst parents, in particular the group with a lower household income to encourage higher paediatric COVID-19 vaccine uptake as well as correct COVID-19 related vaccine misconceptions or vaccine hesitancy, if present.

## Introduction

COVID-19 has had an immense impact on human health worldwide, with close to 600 million confirmed infections worldwide at the time of writing in August 2022 (1). While acute infections have claimed more than 6 million lives, post-acute sequelae of SARS-CoV-2 infection has also had both health and economic impacts. On 31st December 2020, the World Health Organisation (WHO) approved the emergency use of COVID-19 vaccines for adults during the COVID-19 pandemic (2). Despite the availability of the COVID-19 vaccines, vaccine hesitancy remained prevalent in many countries, limiting the success of COVID-19 vaccination programmes worldwide (3–6). This contributed to the COVID-19 pandemic and the advent of more potential virus mutant populations. By December 2021, the Singapore Ministry of Health had also approved the use of Paediatric Dose Pfizer-BioNTech/Comirnaty vaccine for children aged 5–11 years (7). However, the uptake of paediatric COVID-19 vaccination was significantly slower compared to the adult and adolescent COVID-19 vaccination drive (8). This was a worrying trend as unvaccinated children were found to be twice as likely to get hospitalised as compared to vaccinated children (9).

This trend was also observed in the United States of America (USA), where vaccinated adults were less likely to consent to have their children vaccinated against COVID-19 (10). A Bangladeshi study showed that parental vaccine hesitancy could be as high as 42.8% (11). Other Asian countries also demonstrated that parental hesitancy relatively lower at 10.8%–11.8%, in Taiwan and Vietnam respectively (12, 13). Studies in the United Kingdom (UK) have shown that higher social media and digital device usage among parents have been associated with greater hesitancy in vaccinating their children against COVID-19 (14). Additionally, social media use could also lead to a potentially higher likelihood of exposure to COVID-19 misinformation (15). A survey on the use of social media in Singapore estimated that 79.1% the population used some form of social media, wherein the more popular choices of social media were YouTube, Facebook and Instagram (16). Hence, social media usage could have a significant influence on COVID-19 vaccine hesitancy amongst Singaporean parents (17).

In this study, we aimed to compare the social and demographic features of parents with and without hesitancy towards vaccines administration in their offspring. Secondly, we also aimed to determine the impact of social media and device usage by parents in Singapore on COVID-19 vaccine hesitancy. Using the risk factors associated with vaccine hesitancy in parents found in our study, we developed a multinomial logistic regression model to predict COVID-19 vaccine hesitancy amongst parents.

## Materials and methods

A prospective, anonymous and voluntary electronic survey modified from “Measuring vaccine hesitancy: The development of a survey tool” was used (18, 19) (Supplementary Table).

### Study setting and sample

The survey was conducted between 12 November 2021 and 5 March 2022 through FormSG, a secure electronic platform. The study was publicised on the official social media platforms of the National University Hospital of Singapore (NUH) and disseminated *via* email to hospital staff. Posters with QR codes to access the electronic survey were also placed strategically around the hospital, including the Paediatrics and Obstetrics wards and outpatient clinics. Respondents had to be at least 21 years old and be parents. No compensation was provided to the study participants for participating in the study.

### Ethics approval

This study was approved by the National Healthcare Group Domain Specific Ethics Board (NHG DSRB Domain B, Reference No. 2021- 00900) in Singapore.

### Data collection and analysis

Demographic data of respondents and their children was obtained. Time spent on social media (including Facebook, YouTube, Twitter, Weibo, Instagram) and total device usage per week were self-reported by the participants, and then classified as either high or low usage; high usage being defined as more than 12 h per week of social media use and/or 6 h per day of device use. Vaccine hesitancy was a self-assessed variable. Results were analyzed using Chi-square for categorial data and regression analysis was also performed with Statistical Programme for Social Sciences (SPSS), IBM version 27. Statistical significance was defined to be 2-sided when *p* < 0.05.

## Results

### Demographics of study participants

Table 1 shows the demographic data of the 628 parents who participated in the survey. More than two thirds (69.1%) were mothers, with a median of 2 children. Majority (90.1%) had at least pre-university education and were married (90.6%). The median family income bracket of participants were $4,000–$9,999 Singapore dollars per month (1 USD = 1.35 Singapore dollars), which corresponded to the median income of the Singaporean household (Department of Statistics, Singapore). More than two thirds (72.1%) of the respondents were Singaporeans. The modal housing types were 4 to 5 room Housing Development Board (HDB) flats (i.e., public housing) or executive condominiums. With regards to the parents' COVID-19 vaccine status, majority (99.2%) had received at least a dose of the COVID-19 vaccine. 61.4% had at least 1 child eligible for paediatric COVID-19 vaccine and 27.6% had at least 1 adolescent eligible for vaccine. Social media use in the respondents were reported to be 1 to 12 h per week with 1 to 6 h per day on digital devices. They also spent a mode of less than an hour on television every day (Table 2).

**Table 1:** Demographics of participants and comparison of the vaccine hesitant vs. non-hesitant parents.

Demographics	Parents surveyed (*n* = 628)	Vaccine hesitant (*n* = 208)	Not vaccine hesitant (*n* = 420)	*χ*^2^-value	*p*-value
Age (mean ± SD), years	39.1 ± 6.7	39.9 ± 7.02	38.7 ± 6.54	–	0.045
Gender (%)					
Female	434 (69.1%)	132 (63.5%)	302 (71.9%)	4.645	0.031
Male	194 (30.9%)	76 (36.5%)	118 (28.1%)		
No. of children (%)					
1	156 (24.8%)	49 (23.6%)	107 (25.5%)	1.765	0.881
2	315 (50.2%)	110 (52.9%)	205 (48.8%)		
3	143 (22.8%)	45 (21.6%)	98 (23.3%)		
4 or more	14 (2.2%)	4 (1.9%)	10 (2.4%)		
No. of children under age 12 (%)					
0	459 (73.1%)	147 (70.7%)	312 (74.3%)	4.966	0.420
1	85 (13.5%)	30 (14.4%)	55 (13.3%)		
2	59 (9.4%)	20 (9.6%)	39 (9.3%)		
3	22 (3.5%)	10 (4.8%)	12 (2.9%)		
4 or more	3 (0.5%)	1 (0.5%)	2 (0.5%)		
No. of children under age 6 (%)					
0	243 (38.7%)	70 (33.7%)	173 (41.2%)	4.624	0.593
1	180 (28.7%)	65 (31.3%)	115 (27.4%)		
2	138 (22.0%)	51 (24.5%)	87 (20.7%)		
3	53 (8.4%)	18 (8.7%)	35 (8.3%)		
4 or more	14 (2.2%)	4 (1.9%)	10 (2.4%)		
Education level (%)					
Primary school or lower	9 (1.4%)	4 (1.9%)	5 (1.2%)	25.407	<0.001
Secondary school/ Institute of technical education	53 (8.4%)	31 (14.9%)	22 (5.2%)		
Junior College / Polytechnic	94 (15.0%)	35 (16.8%)	59 (14.0%)		
University Degree	317 (50.5%)	104 (50%)	213 (50.7%)		
Master's Degree or above	155 (24.7%)	34 (16.3%)	121 (28.8%)		
At least pre-university education					
No	62 (9.9%)	35 (16.8%)	27 (6.4%)	16.904	<0.001
Yes	566 (90.1%)	173 (83.2%)	393 (93.6%)		
Area of work (%)					
Healthcare	200 (31.8%)	43 (20.7%)	157 (37.4%)	–	–
Financial	63 (10.0%)	17 (8.2%)	46 (11.0%)		
Service Industry	39 (6.2%)	14 (6.7%)	25 (6.0%)		
Manufacturing	40 (6.4%)	16 (7.7%)	24 (5.7%)		
Education	83 (13.2%)	26 (12.5%)	57 (27.4%)		
Energy and Infrastructure	22 (3.5%)	8 (3.8%)	14 (3.3%)		
Biotechnology	9 (1.4%)	3 (1.4%)	6 (1.4%)		
Information and communication technologies	54 (8.6%)	24 (11.5%)	30 (7.1%)		
Transport	18 (2.9%)	12 (5.8%)	6 (1.4%)		
Freelance/Self-employed	33 (5.3%)	18 (8.7%)	15 (3.6%)		
Housewife/home maker	66 (10.5%)	26 (12.5%)	40 (9.5%)		
Unemployed	1 (0.2%)	1 (0.5%)	0 (0%)		
Religion (%)					
Buddhism	126 (20.1%)	49 (23.6%)	77 (18.3%)	7.865	0.345
Catholic	43 (6.8%)	16 (7.7%)	27 (6.4%)		
Christianity	147 (23.4%)	36 (17.3%)	111 (26.4%)		
Hindu	41 (6.5%)	15 (7.2%)	26 (6.2%)		
Muslim/Islam	119 (18.9%)	38 (18.3%)	81 (19.3%)		
Taoism	25 (4.0%)	9 (4.3%)	16 (3.8%)		
No religion	124 (19.7%)	44 (21.2%)	80 (19.0%)		
Others	3 (0.5%)	1 (0.5%)	2 (0.5%)		
Place of Birth (%)					
ingapore	453 (72.1%)	154 (74.0%)	299 (71.2%)	8.373	0.137
Malaysia	61 (9.7%)	19 (9.1%)	42 (10%)		
India, Sri Lanka, Pakistan	38 (6.1%)	13 (6.3%)	25 (6.0%)		
China, Taiwan, Hong Kong, Japan	24 (3.8%)	12 (5.8%)	12 (2.9%)		
Non- Malaysian SEA	36 (5.7%)	8 (3.8%)	28 (6.7%)		
ANZ, USA, Europe, South America	16 (2.5%)	2 (1.0%)	14 (3.3%)		
For non-Singaporean, median no. of years residence in Singapore (mean ± SD), years	15.7 ± 8.3	16.2 ± 8.1	14.6 ± 8.4	1.189	0.237
Experience studying or working abroad (%)					
Yes	213 (33.9%)	64 (30.8%)	149 (35.5%)	1.375	0.24
No	415 (66.1%)	144 (69.2%)	271 (64.5%)		
Average reported monthly family income in Singapore dollars					
Below $4,000	59 (9.4%)	26 (12.5%)	33 (7.9%)	18.658	<0.001
$4,000–$9,999	260 (41.4%)	99 (47.6%)	161 (38.3%)		
$10,000–$14,999	160 (25.5%)	54 (26.0%)	106 (25.2%)		
$15,000–$19,999	68 (10.8%)	13 (6.3%)	55 (13.1%)		
$20,000 or above	81 (12.9%)	16 (7.7%)	65 (31.3%)		
Income bracket					
Below Median ($4,000–$9,999)	319 (50.8%)	125 (60.1%)	194 (46.2%)	11.542	0.003
Above median	309 (49.2%)	83 (39.9%)	226 (53.8%)		
Type of Housing					
Government rental housing	28 (4.5%)	12 (5.8%)	16 (3.8%)	14.029	0.015
Government public 1- 3 rooms flat	49 (7.8%)	12 (5.8%)	37 (8.8%)		
Government public 4-5 rooms flat/ executive condominium	361 (57.5%)	131 (63.0%)	230 (54.8%)		
Maisonette	10 (1.6%)	3 (1.4%)	7 (1.7%)		
Private Condominium/ Landed Property	177 (28.2%)	47 (22.6%)	130 (31.0%)		
Others	3 (0.5%)	3 (1.4%)	0 (0%)		
Marital Status (%)					
Single	5 (0.8%)	2 (1.0%)	3 (0.7%)	1.709	0.635
Married	608 (96.8%)	203 (97.6%)	405 (96.4%)		
Divorced	13 (2.1%)	3 (1.4%)	10 (2.4%)		
Separated	2 (0.3%)	0 (0%)	2 (0.5%)		
COVID-19 Vaccination status of participant (%)					
At least one dose	623 (99.2%)	203 (97.6%)	420 (100%)	10.177	0.001
Unvaccinated	5 (0.8%)	5 (2.4%)	0 (0%)		

**Table 2:** Survey results.

Questions	Parents surveyed (*n* = 628)	Vaccine hesitant (*n* = 208)	Not vaccine hesitant (*n* = 420)	*χ*^2^-value	*p*-value
Daily reported average use of digital devices (%)					
Less than an hour	14 (2.2%)	9 (4.3%)	5 (1.2%)	7.850	0.097
Between 1 and 6 h	340 (54.1%)	115 (55.3%)	225 (53.6%)		
Between 6 and 12 h	209 (33.3%)	62 (29.8%)	147 (35%)		
Between 12 and 18 h	45 (7.2%)	14 (6.7%)	31 (7.4%)		
More than 18 h	20 (3.2%)	8 (3.8%)	12 (2.9%)		
Low vs. high usage of digital devices					
Low	354 (56.4%)	124 (59.6%)	230 (54.8%)	1.332	0.248
High	274 (43.6%)	84 (40.4%)	190 (45.2%)		
Weekly reported amount of time spent on social media (%)					
Less than 1 h	100 (15.9%)	39 (18.8%)	61 (14.5%)	5.073	0.280
Between 1 and 12 h	410 (65.3%)	138 (66.3%)	272 (64.8%)		
Between 12 and 24 h	59 (9.4%)	13 (6.3%)	46 (11.0%)		
Between 24 and 72 h	39 (6.2%)	12 (5.8%)	27 (6.4%)		
More than 72 h	20 (3.2%)	6 (2.9%)	14 (3.3%)		
Low vs. high amount of time spent on social media (%)					
Low	510 (81.2%)	177 (85.1%)	333 (79.3%)	3.078	0.079
High	118 (18.8%)	31 (14.9%)	87 (20.7%)		
Daily reported amount of time spent watching television (%)					
Less than 1 h	387 (61.4%)	136 (65.4%)	251 (59.8%)	4.706	0.319
Between 1 and 6 h	229 (36.5%)	66 (31.7%)	163 (38.8%)		
Between 6 and 12 h	7 (1.1%)	4 (1.9%)	3 (7.1%)		
Between 12 and 18 h	3 (0.5%)	1 (0.5%)	2 (0.5%)		
More than 18 h	2 (0.3%)	1 (0.5%)	1 (0.2%)		
Personally know someone with a bad reaction to the vaccine					
No	338 (53.8%)	89 (42.8%)	249 (59.3%)	15.233	<0.001
Yes	290 (46.2%)	119 (57.2%)	171 (40.7%)		
All things considered, how much are you able to trust your child's doctor					
Distrust	4 (0.6%)	4 (1.9%)	0 (0%)	45.606	<0.001
Somewhat trust	250 (39.8%)	117 (56.3%)	133 (31.7%)		
Fully trust	374 (59.6%)	87 (41.8%)	287 (68.3%)		

### Characteristics of vaccine-hesitant parents

Two thirds of the respondents (66.9%) identified as not vaccine hesitant. A third (27.2%) considered themselves somewhat hesitant towards childhood vaccination.

Mothers were less vaccine hesitant than fathers (71.9% vs. 63.5%, *p* < 0.05; Table 1). Vaccine hesitancy was significantly associated with lower education level, lower household income as well as being unvaccinated for COVID-19. There was no difference with high usage of social media and device use between both groups.

Majority (91.6%) acknowledged that they could make shared parental decisions with their children's doctor and discuss their concerns openly. More than half (59.6%) trusted their child's doctor fully while 39.8% reported that they somewhat trusted their doctor. Childhood vaccine hesitant parents were less likely to trust their child's doctor compared to parents who were not vaccine hesistant (41.8% vs. 68.3%, *p* < 0.001; Table 2).

**Figure 1:**
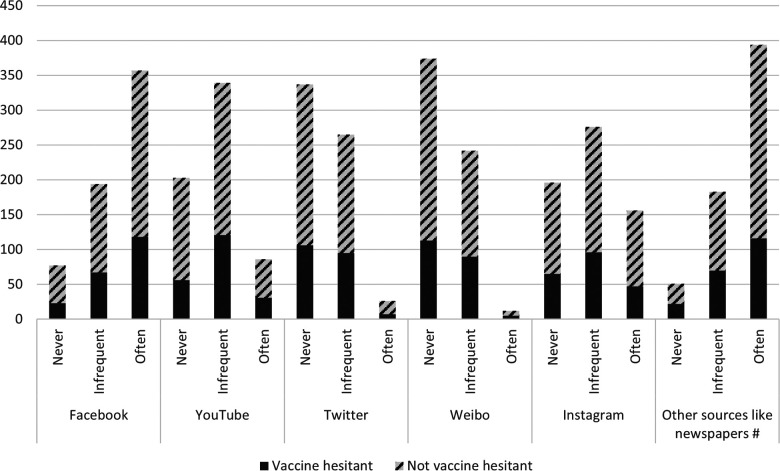
Frequency of use of social media, newspapers or other print materials and television to obtain information about COVID-19 for all parents.

Parents reported that the more common sources of COVID-19 related updates were from printed media (i.e., newspapers) (62.7%) and then Facebook (56.8%) and Instagram (24.8%) (Figure 1). Twitter and Weibo were the less likely to be used among respondents to obtain updates. YouTube and Instagram were also used infrequently.

There was no difference between vaccine hesitant and non-hesitant parents in their use of social media to obtain information about the COVID-19 pandemic. However, parents who were not vaccine hesitant were more likely to use other sources such as newspapers frequently to obtain COVID-19 related information as compared to vaccine hesitant parents. (66.2% vs. 55.8%, *p*  <  0.01).

Vaccine hesitant parents who had an education level of pre-university level and below, were less likely to use printed media to obtain COVID-19 related information. (80.6% vs. 91.0%, *p* < 0.05).

Almost half (46.2%) of respondents knew someone with a bad reaction to the vaccine personally. They were also more likely to be vaccine hesitant (57.2% vs. 40.5%, *p* < 0.01) as compared to parents who did not personally know anyone with a bad reaction to the vaccine.

Using the various associated risk factors identified in Table 3, a logistic regression model was formed. Trust in their child's doctor was found to be the most significant factor influencing a parent's decision in childhood vaccination. Knowing someone with a bad vaccine reaction, gender of respondents and housing type were not statistically significant when the regression model was used.

**Table 3:** Regression model used to determine vaccine hesitancy.^a^

Model	Unstandardized Coefficients		Standardized Coefficients *β*	*t*-value	Significance	Correlations			Collinearity Statistics	
	B	Standard Error				Zero-order	Partial	part	Tolerance	VIF
(Constant)	1.761	.387	–	4.552	<.001	–	–	–	–	–
Number of years studied overseas	−0.011	0.005	−0.155	−2.158	.032	−0.137	−0.169	−0.152	.959	1.043
Do you know of anyone who has had a bad reaction to the vaccine?	.041	0.067	.045	.621	.536	.109	.049	.044	.935	1.069
All things considered, how much do you trust your child's doctor?	−0.260	.069	−0.280	−3.797	<.001	−0.322	−0.288	−0.268	.915	1.092
Gender	.096	.070	.099	1.382	.169	.045	.109	.097	.971	1.030
Have you received your COVID-19 jab?	−0.541	.302	−0.128	−1.791	.075	−0.169	−0.141	−0.126	.968	1.033
Type of housing	.028	.030	.073	.930	.354	−0.052	.074	.066	.803	1.245
Family monthly income	−0.076	.032	−0.190	−2.362	.019	−0.230	−0.184	−0.167	.771	1.298
Education level	−0.095	.052	−0.136	−1.847	.067	−0.200	−0.145	−0.130	.921	1.086

VIF, Variance inflation factor.

^a^ Vaccine hesitancy for childhood vaccines among parents was the dependent variable.

When the variables of gender, household income status, vaccine status were further analysed with a multinomial logistic regression model, vaccine hesitancy in a parent could be predicted with a 70% accuracy, and non-vaccine hesitancy with a 92.4% accuracy.

## Discussion

This is a multi-ethnic study done in Southeast Asia which lends a unique perspective of parental values in vaccinating their children against COVID-19. In our study, vaccine hesitancy in parents was found to be correlated with being a father, lower household income levels, parents' own unvaccinated status with a 92.4% accuracy. These associations may allow healthcare workers in identifying and tailoring their approach in making shared parental decision making with parents who may be vaccine hesitant.

A key finding by our study which could impact the rate of vaccination uptake amongst the paediatric population is the importance of parental trust in their children's primary healthcare provider. This was identified as the most significant factor contributing to the COVID-19 vaccine uptake amongst parents in our study. In fact, the COVID-19 National Preparedness Collaborators had identified that a high level of trust is not only associated with higher vaccine uptake but also lower levels of infection rates during the pandemic (20). We recommend that healthcare providers opportunistically discuss vaccine safety during routine healthcare encounters, to guide parents in making informed decisions regarding COVID-19 vaccination for their children.

Our findings suggest that parents with lower household incomes were more comfortable using social media as opposed to print material as a sole source of information. In contrast, despite the relatively high usage of social media and digital devices in Singapore, parents with a higher level of education preferred to obtain information regarding COVID-19 vaccines from newspaper and print material rather than social media. Thus, to ensure that critical information on vaccine safety is disseminated across all socioeconomic strata, the medium through which information is provided is important. Our findings suggest that a multi-pronged approach including different media forms directed at different socioeconomic groups would be necessary for a successful vaccine information campaign.

Our findings of the influence of social media and device usage contradicts that of findings reported by other studies (12, 13, 20–24). In our study, high usage of digital devices and social media use did not influence the vaccination choices made by parents in Singapore. These findings are contrary to the findings reported by the European and American adults as well as Japanese parents. One explanation for this is that supplementing social media usage with consumption of print media decreased vaccine hesitancy in the parents in our study, thus highlighting that it may not be the usage of social media but the exclusive reliance on social media for health information that leads to high vaccine hesitancy. Another explanation is that it may not be the usage of formal or informal sources of information, but rather than level of trust that an individual puts in the sources, which is more highly predictive of vaccine hesitancy (24). Hence, the availability of reliable vaccine safety information through mainstream news media could play an additive role in supplementing social media users with information that encourages parents to vaccinate their children, thus increasing COVID-19 vaccine uptake.

Due to our convenient sampling of parents from the hospital, there was an over-representation of those working in healthcare, as well as those who consult medical professionals for health issues. In addition, we expect that unvaccinated parents may be under-represented, as they may be less inclined to fill up a survey about vaccines. Taken together, our study may under-estimate vaccine hesitancy among parents in Singapore. Finally, as with self-reported surveys, there is a possibility of recall bias resulting in over- or underestimation of the events reported. A bigger study including other Asian societies with diverse socio-economic backgrounds will allow for further exploration of the problem of vaccine hesitancy, which has been identified by the World Health Organisation to be a key threat to human health.

In conclusion, newspaper and print media with reliable vaccine safety information appears to be a useful tool in educating parents about vaccine safety and efficacy, especially among highly educated parents in Singapore. Trust in the child's doctor was found to be a significant factor in childhood vaccine hesitancy.

## Data Availability

The raw data supporting the conclusions of this article will be made available by the authors, without undue reservation.
