# Supplementary material for: Predicting vaccine hesitancy among parents towards COVID-19 vaccination for their children in Singapore
Source: Front Pediatr. 2022 Oct 10;10:994675. doi: 10.3389/fped.2022.994675 (PMC9589407; doi:10.3389/fped.2022.994675)
Supplement: Supplementary file 1 [file Table1.docx]

Supplementary Material

# Supplementary Table. Survey questions, adapted from Larson et al., 2015.

| **Category** | **Questions** |
| --- | --- |
| **Demographic Data** | - What is your gender? - What is your year of birth? - How many children do you have?   - How many of them are above 6 years old?   - How many of them are above 12 years old? - What is your education level? - What is your area of work? - What is your religion? - Where is your place of birth? - If you were not born in Singapore, how many years have you resided here? - Have you worked or studied overseas? If so, how many years were you working/ studying overseas? - What is your family monthly income in S$? - What type of housing do you live in? - What is your marital status? |
| **Change in routine due to COVID-19 pandemic** | - Did you have a change from the usual work schedule from the office to home during the period of lockdown? - Did you work from home during the periods of lockdown? |
| **COVID-19 vaccination status and vaccination behavior** | - Have you received your COVID-19 jab? If no, what are the reasons why you have not received a COVID-19 vaccine? - Are you fully vaccinated against COVID-19? If not, what is the reason? |
| **Digital device and media use** | - What is your daily average use of digital devices for this week? - What is the total amount of time you spent on social media this week? - How much time on average do you spend watching TV per day? - How often if ever do you get news and updates on COVID-19 outbreaks from each of the following places? (Facebook, YouTube, Twitter, Weibo, Instagram, others such as newspapers) - Please tell us how much of what you know about COVID-19, if anything, comes from (Facebook/ Twitter or WhatsApp messages, Newspapers or other print material like magazines/ health leaflets, Television)? |
| **Vaccine safety and efficacy beliefs** | - Do you agree with the following beliefs about vaccine safety and efficacy? - Children get more vaccines than are good for them. - Many of the illnesses vaccines prevent are severe. - It is better for children to develop immunity by getting sick than by getting a shot. - It is better for children to get fewer vaccines at the same time. - How concerned are you that your child might have a serious side effect from a vaccine? - How concerned are you that any one of the childhood vaccines may not be safe? - How concerned are you that a vaccine might not prevent the disease? - Do you know anyone who has had a bad reaction to a vaccine? |
| **Overall attitude and trust** | - Have you (or would you intend to) ever delayed having your child get a vaccine for reasons other than illness or allergy? - Do you agree that the recommended vaccine schedule is (or would be) a good idea for your child? - Do you agree to the following statements? - It’s my role as a parent to question vaccines. - If you had another infant today, would you want him/her to get the recommended shots? - Overall, how hesitant about childhood vaccines would you consider yourself to be? - Which of the following statements reflect your general attitude and trust towards vaccines? - The only reason I would have my child get vaccines is do that they can enter day-care or school. - I trust the information I receive about vaccines. - Are you able to openly discuss your concerns about vaccines with your child’s doctor? - All things considered, how much do you trust your child’s doctor? - Would you give the COVID-19 vaccine (mRNA) to your teenager? - Would you give the COVID-19 vaccine (mRNA) to your child between 6-11 years old (if any)? - If HAS/MOH licenses a vaccine for use in children ,12 years old, would you give the COVID-19 vaccine (mRNA) to your child between 6- 11 years? - Do you agree with the following statements? - My government has managed the current pandemic well. - My country health care system/ health department has managed the current pandemic well. - Which have been your main sources of information about the safety and effectiveness of the COVID-19 vaccines? (Government and Health Department information, Television and radio, YouTube and bloggers, social media and large groups, Friends and small groups) - Which of the following vaccine types do you think is safest? (Inactivated vaccines, non-replicating viral vectors, mRNA, Protein subunit, DNA) - Which of the following vaccine types do you think is most effective? (Inactivated vaccines, non-replicating viral vectors, mRNA, Protein subunit, DNA) - If you could choose any vaccine in the world which vaccine, would you select and why? |
